# Mechanical interlocking of cotton fibers on slightly textured surfaces of metallic cylinders

**DOI:** 10.1038/srep25403

**Published:** 2016-05-09

**Authors:** Youqiang Zhang, Yu Tian, Yonggang Meng

**Affiliations:** 1 State Key Laboratory of Tribology, Tsinghua University, Beijing 100084, China; 2 School of Mechanical and Electrical Engineering, Key Laboratory of Modern Agricultural Engineering, Tarim University, Alar 843300, China

## Abstract

Mechanical interlocking is widely applied in industry and general lives of human beings. In this work, we realized the control of locking or sliding states of cotton fibers on the metal surfaces with slightly different textures through traditional machining. Three types of sliding states, i.e., locking, one-way sliding, and two-way sliding have been achieved. It is found that the locking or sliding of the cotton fibers on the metallic cylinder depends on the friction coefficient and the ratio of cotton fiber diameter, 2r, to the height of the rough peaks, h, of metal surfaces. When the critical ratio h/r exceeds 1, the cotton fibers could tightly attach to the metallic surface through mechanical interlocking. This work provided a convenient and universal method for the control of interlocking or sliding of fiber-based materials on textured surfaces.

Surface texture of organisms attracts more and more interests of researchers^1^,^2^,^3^. On one hand, the surface texture of various organisms is crucial for their adaptation to the natural environment^3^. On the other hand, numerous excellent tribological properties of the nature inspire people to design mechanical structures and surface textures that could increase or reduce friction to be used in industries^1^,^4^,^5^. Mechanical interlocking is a successful application inspired from the nature. For instance, cocklebur (Xanthium strumarium L.) is an annual weed found in temperate and subtropical regions around the world; its fruit readily attaches to the hair and fur of animals for seed dispersal^6^,^7^,^8^. The contact separation force of fruit burrs in cocklebur for seed dispersal exhibits mechanical interlocking^9^.

Many animals rely on their claws and the adhesive pads on their feet to prey foods or climb various surfaces during their movement^3^,^10^. In flies and geckos, the adhesion interaction of their feet with substrates is controlled by the van der Waals force based adhesion and friction with hierarchical structures of their setae; such hierarchical structure makes it adaptive to different roughness of substrates^11^,^12^,^13^. The stability of the unidirectional adhesion interaction between setae and a solid surface depends on the mechanism of anisotropic structures of setae arrays^14^.

Different from those setae structures on feet of creatures, numerous fibrous surface structures can attach to other substrate surfaces, these structures improve the binding force between fibers and other substrates^15^,^16^. Wool is probably the first fiber to be used for the production of cloth; wool fibers comprise cuticle cells (scales), and their horn surfaces contain small cracks that provide asperities with which the scales of wool fibers can interact when they slide on them^17^,^18^. Cotton fibers are the most common natural fibers; their frictional behavior as they attach to other substrates is largely dependent on their properties, such as fiber length, fiber fineness, and the twisted-ribbon shape along the fiber length^19^,^20^,^21^.

Mechanical engagement or mechanical interlocking is widely known as a universal adhesion mechanism used in industry. However, while macroscopic mechanical interlocking or anisotropic properties of natural or artificial adhesion have been widely studied in former researches, controlling the unidirectional or bidirectional sliding between a buddle of fibers and another solid surface has not been realized yet. Therefore, in this study, the sliding behavior of a cotton fiber on metallic surfaces with different surface morphologies achieved from traditional turning and rough polishing. The mechanical interlocking was used to explain the effect of surface morphology on significantly different sliding friction behaviors.

## Results and Discussion

### Friction test

**Figure 1:**
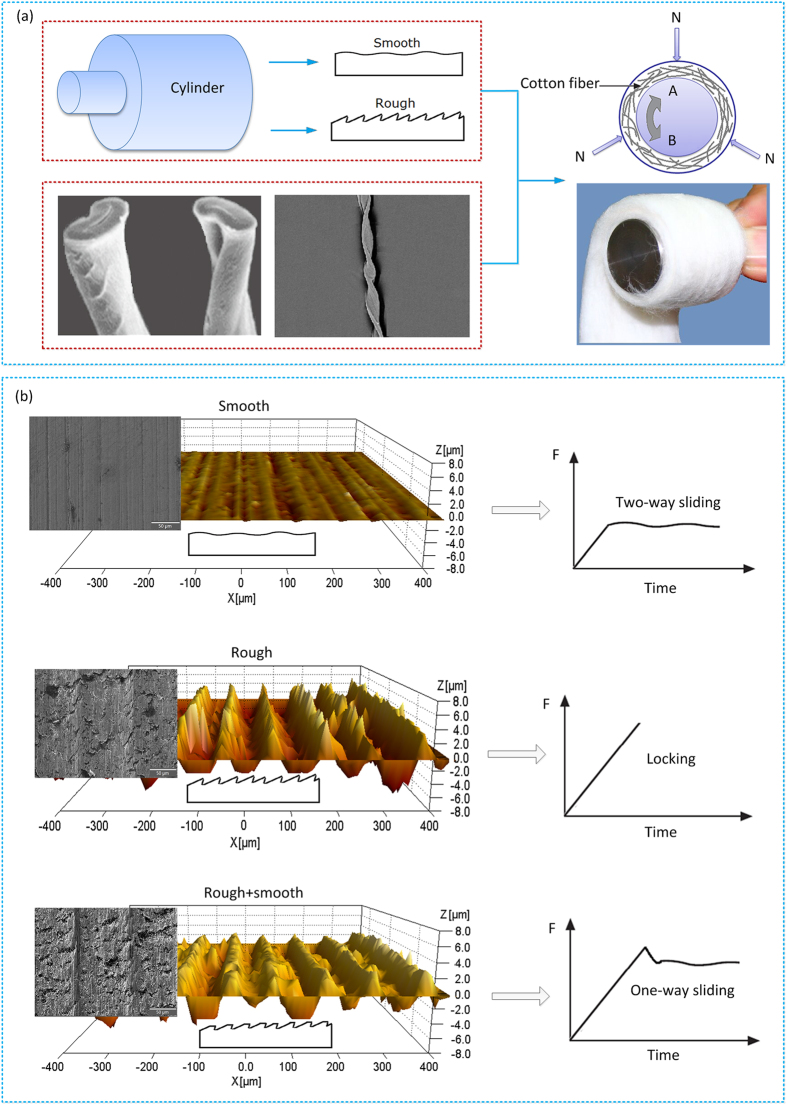
Concept and fabrication of friction between the cotton fiber and the metallic surface. (**a**) The features morphology of cotton fiber and schematic diagram of the sliding test between combed cotton and metallic surface. (**b**) Three different surface morphologies (smooth/rough/rough & smooth) and the schematic diagram of their respective friction forces.

Cotton fibers usually consist of the network of pores with different sizes or capillary spaces surrounded by numerous fibrils in different layers. Macroscopically, cotton fibers are twisted ribbons along their length and have a kidney-shaped cross section^19^. The layered features and the corresponding cross section morphology of a single cotton fiber were shown in Fig. 1(a).

The SEM and three dimensional surface morphologies of the three different surfaces of metallic cylinders were shown in Fig. 1(b). Friction behaviors of the cotton fiber layer sliding the metal surface with different surface morphologies were tested. With a certain torque exerted on the cylindrical specimens, the following three modes of sliding could be identified.

1. Two-way sliding was observed with smooth metal surface.
2. Two-way locking occurred on rough metal surface with relatively deep groove and a scaly gullet growth.
3. One-way sliding and one-way locking occurred on rough surface that was slightly polished by sandpaper to reduce asperity height and partially removed scaly gullet.

**Figure 2:**
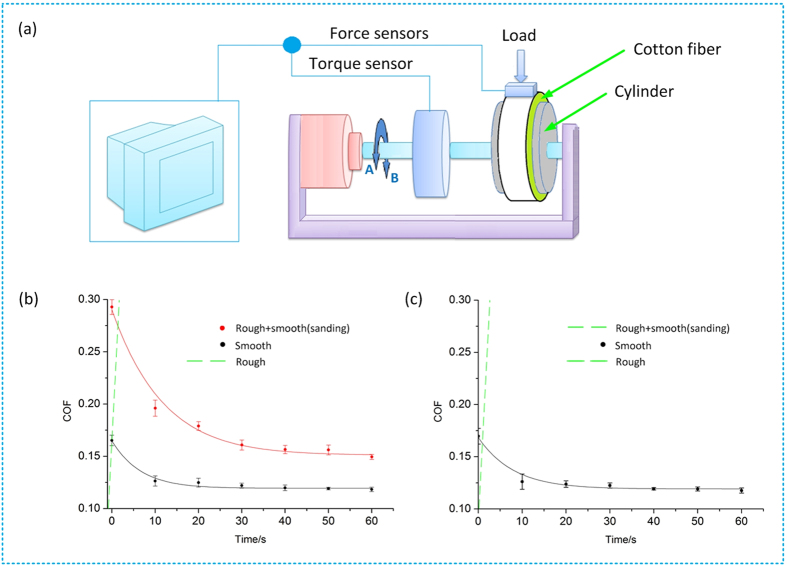
The coefficients of friction over time. (**a**) Schematic diagram of the friction test on the cotton fiber and metallic surface. (**b**,**c**) Cotton fiber coming into contact with the grinded surface, sanded surface, and turned surface for directions A and B, respectively.

A fiber-metal friction test device was established to characterize the sliding friction behavior of cotton fiber layer on metal surfaces as illustrated in Fig. 2(a). Figure 2(b,c) presented the friction coefficients between the cotton fibers and different textured surfaces of the metallic cylinder for different friction directions A and B. The friction coefficient was relatively low between the grinded surface and cotton fibers and was approximately consistent for directions A and B.

Figure 2(b,c) indicated that the sliding friction coefficient between the cotton fibers and rough surface increased sharply and entered a locked state in both directions. For the sanded rough surface, sliding was possible only in direction A while locked in direction B. The sanded surface achieved a higher friction coefficient than the smooth surface for direction A. Figure 2(b,c) also showed that when sliding was observed in directions A or B, the coefficient of friction quickly decreased within 20 seconds and then tended to be stable for a long time.

### The influence of surface topographies on mechanical interlocking

**Figure 3:**
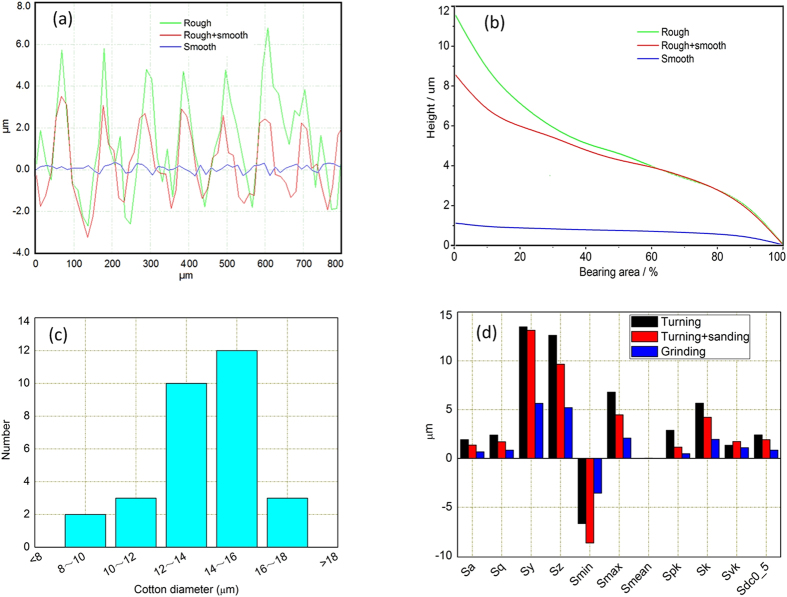
Schematic illustrating the relative dimension between the cotton fiber and the rough peak of the cylindrical surface. (**a**) Cross-section curve of the three-dimensional morphologies of the specimens. (**b**) Curve of the bearing area of the specimens. (**c**) Diameter distribution of the section of the combed cotton. (**d**) Surface topography parameters of turning, turning & sanding, and grinding. (S_a_, Roughness Average; S_q_, Root Mean Square; S_y_, Peak-Peak Height; S_z_, Ten Point Height; S_min_, Max Valley Depth; S_max_, Max Peak Height; S_mean_, Mean Value; S_pk_, Reduced Summit Height; S_k_, Core Roughness Depth; S_vk_, Reduced Valley Depth).

The cross-section of metallic cylinder surface is similar to a V-shaped groove. Figure 3(a) showed one of the cross-section profiles of the specimens. The V-shaped groove was approximately 100 μm in width, and 9 μm in height for the turned surface (rough surface), 4.5 μm in height for the turned & sanded surface (sanded surface), and 1.5 μm in height for the grinded surface (smooth surface). These results were supported by the curve of the bearing area, as shown in Fig. 3(b).

Geometrically, a cotton fiber is like a twisted ribbon along its length and features a kidney-shaped cross section with a nearly circular transverse surface^19^,^22^. The diameter distribution of the section of the combed cotton was shown in Fig. 3(c). The average diameter was about 14 μm. Its typical characteristics are approximately 20–40 twists/cm.

The surface topography parameters of rough, sanded, and smooth surfaces are presented in Fig. 3(d). The S_y_ and S_z_ values of the smooth surface were all less than 7 μm similar to the radius of cotton fibers. And that of the other surfaces were greater than 7 μm. The slight difference between the rough and sanded surfaces was accurately revealed by the distribution of S_y_, S_z_, and S_pk_. The S_min_ and S_vk_ values of the sanded surface were greater than that of the rough surface. Scratching the peak valley may be grinded in the process of sanding after turning. The peak tips were severely worn down in the sanding process, as indicated by the distribution of S_max_ and S_pk_. The cotton fiber was embedded in the V-shaped groove of rough surface under normal force. When the height of the V-shaped groove was reduced from 9 μm to 4.5 μm, the cotton fiber could not be fully embedded. When the height of the V-shaped groove was less than 1.5 μm, the fiber could not be embedded at all.

The friction force was proportional to the actual contact area, which was supported by the experimental results of numerous studies^15^,^19^,^23^. The friction force between the cotton fibers and rough surface was greater than the resulting of Coulomb friction. The irregular asperity distribution increased the resistance to fiber sliding in the V-shaped groove. Therefore, the cotton fibers were stuck on the rough surface. The sliding in the cotton fibers layer required overcoming the extra-high resistance. A proper amount of restriction led to the mechanical interlocking between the cotton fibers and rouge surface of the cylinders.

When the asperities were even, such as in the smooth surface, the height of the asperities was relatively small, and the number of embedded cotton fibers decreased. This condition significantly reduced the interlocking between fibers and rough peaks. The small resistance in this case required a low torque to slide the fibers on metal surfaces in both directions.

### Model of mechanical interlocking

**Figure 4:**
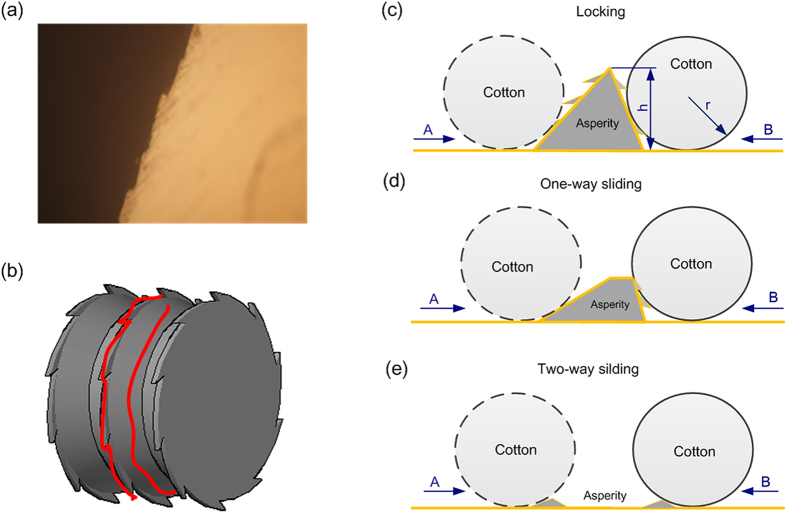
Schematic illustrating the geometrical configurations of the surface morphology. (**a**) Serrated morphology of the turned surface under an optical microscope (500×). (**b**) Model of mechanical interlocking. (**c**–**e**) Illustrations of the geometrical models of mechanical interlocking through turning machining, turning & sanding, and grinding, respectively.

In the present study, we achieved a serrated morphology on the surface of the turned specimens, as shown in Fig. 4(a). The surface was covered with jagged burrs with geometric angle. The cotton fibers adhered closely to the rough surface under normal load when the cotton fibers were pressed on the rough surface. The rough peak was similar to the ratchet mechanism and led to mechanical interlocking. The model of mechanical interlocking was illustrated in Fig. 4(b).

Cotton fibers are sensitive to slight differences in surface morphology. The slip-styles of the cotton fiber to the metallic cylinder were controlled by the relative dimension between the cotton fiber and rough peak. Table 1 illustrates the test results of friction between the cotton fibers and the differently textured metal surfaces. The friction coefficient increased with the increase of the height of the rough peak. The two-way sliding occurred when the value of h/r (r, the average radius of the circular section of cotton fibers, h, the height of the rough peak, Fig. 4(c)) was less than 1 because the height of the rough peak was not sufficient to produce mechanical interlocking.

The geometrical configurations of the cotton fibers and differently textured surfaces of the metallic cylinder can be classified into three modes: locking, one-way sliding, and two-way sliding. The three modes correspond to the cases of rough, sanded and smooth surfaces, as illustrated in Fig. 4(c–e) respectively. The mechanical interlocking led to non-sliding between the cotton fibers and rouge surfaces. The bidirectional mechanical interlocking constantly occurred when the value of h/r was greater than 1 (Fig. 4(c)). Interestingly, after the scaly gullet was partially removed, the mechanical interlocking was eliminated along with the direction of sanding (i.e., direction A). But it was not eliminated with the direction without sanding (direction B). The change of the relative dimension between the fiber diameter and the topography parameters of the metallic cylinder surface could lead to frequently and alternatively occurrence of the mechanical interlocking and two-way sliding. The two-way sliding and bidirectional interlocking randomly happened in the adjacent zone (h/r = 1) of the two-way sliding and locking. Obviously, The two-way sliding inevitable occurs when the value (h/r) was less than 1 (Fig. 4(e)), because there is no mechanical interlocking between the cotton fiber and smooth surface.

## Conclusions

In this study, we successfully fabricated surfaces with different surface roughness (rough, sanded, and smooth) of the metallic cylinders through traditional machining. The locking, one-way sliding, and two-way sliding were realized through the slightly change in the surface morphologies of the metallic cylinder. The friction coefficient represented the locking or sliding of the cotton fibers on the metallic surfaces. The locking or sliding of the cotton fiber on the metallic surface was mainly determined by the relative ratio of the height of the rough peak and the cross-section radius of the cotton fibers. Mechanical interlocking occurred when the value of h/r was greater than 1; it was not observed when the value was less than the 1. The one-way sliding occurred because the roughness was reduced (h/r < 1) and the serrated spikes were removed in the sanding direction. The test results indicated the sensitivity of small fibers to any slightly different surface morphology, and could provide a theoretical and experimental basis for the surface texture design for applications such as textile machinery and spindles for cotton pickers.

## Materials and Methods

### Cylindrical specimens

Generally, the surfaces used in practical engineering are produced through traditional machining (e.g., turning, milling, boring, and grinding). Compared with that of micro/nano manufacturing technologies, the distribution of surface textures obtained via traditional machining is thought to be not so regular and precise. Therefore, the traditional mechanical process is often disregarded in the pursuit of a regular distribution of surface textures. However, traditional manufacturing can usually bring naturally formed cutting ridges which could not be realized with precision micro/nano manufacturing methods. Therefore, in the present study, three different surface textures on the cylindrical specimens (smooth, rough, grinded rough surfaces) were fabricated expediently via traditional machining methods.

Cylindrical specimens with 25 mm in diameter and 35 mm in height were made from 210Cr12 steel (ISO) with a hardness of 269–271 HB. The surface morphologies of the specimens were observed by scanning electron microscope (SEM, FEI Quanta 200 FEG). The three dimensional morphology of the surface were determined by scanning 857 × 638 μm^2^ area using three dimensional surface topography (MICROXAM–3D). The calculations were performed using the scanning probe image processor (SPIP) software, which is the standard program for processing three dimensional surface topography data. To obtain metallic cylinders with different surface morphologies, different machining parameters were adopted and shown in Table 2. After turning, some surfaces were subtly grinded with sandpaper (PEPA P#2400, Struers) along direction A (see Fig. 1(a)).

### Cotton fiber

Cotton fibers are natural cellulose materials obtained from the mature capsules of cotton plants. The cotton fibers in this work were carefully combed into cotton strips (generally called combed cotton). Table 3 listed the parameters of the used cotton fibers in this study.

### Friction Sliding Test

The sliding was carried out by rotating the metal cylinder driven by a servo motor (in Fig. 2(a), A and B represent clockwise and counter clockwise directions, respectively). The winding of fibers on the metal surface and the regulation of radial force were through a clamping mechanism. Each combed cotton strip was entwined tightly around the cylindrical surface of the specimen under normal force. The pressure exerted on the fiber layer was monitored with force sensors. The coefficients of friction were obtained from the normal force (N) and torque (T) as


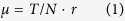


where *r* is the radius of the specimen and *μ* is the coefficient of friction (COF). All the friction tests were conducted at 30% ambient humidity and a room temperature of about 25 °C. The normal load and rotation speed were 30 N and 500 rpm, respectively. The friction coefficients between the cotton fiber and the metallic surface were averaged from six measurements. The specimens were cleaned with acetone before every test.

## Additional Information

**How to cite this article**: Zhang, Y. *et al*. Mechanical interlocking of cotton fibers on slightly textured surfaces of metallic cylinders. *Sci. Rep.* **6**, 25403; doi: 10.1038/srep25403 (2016).

## Figures and Tables

**Table 1:** Switching process of two-way sliding to locking.

h/r	Dir	Coefficient of friction (COF)															
		T1	T2	T3	T4	T5	T6	T7	T8	T9	T10	T11	T12	T13	T14	T15	T16
0.21	A	0.12	0.12	0.11	0.12	0.12	0.12	0.11	0.13	0.12	0.12	0.12	0.12	0.11	0.12	0.11	0.12
	B	0.13	0.12	0.12	0.13	0.13	0.13	0.12	0.14	0.12	0.13	0.14	0.11	0.13	0.14	0.12	0.13
0.64	A	0.15	0.14	0.15	0.15	0.16	0.14	0.15	0.14	0.15	0.16	0.13	0.15	0.15	0.16	0.15	0.15
	B	0.15	0.13	0.14	0.13	0.13	0.15	0.15	0.13	0.14	0.14	0.15	0.12	0.14	0.15	0.13	0.14
0.95	A	0.18	0.17	0.19	0.16	0.17	0.16	0.17	0.16	0.17	0.17	0.16	0.17	NS	0.19	0.18	0.17
	B	0.19	0.18	NS	0.19	0.17	0.19	0.20	0.21	0.21	0.24	0.19	0.20	0.18	NS	0.20	0.21
1.02	A	NS	NS	NS	NS	NS	NS	NS	0.24	NS	NS	NS	NS	NS	0.25	NS	NS
	B	NS	NS	NS	0.25	NS	NS	NS	NS	NS	NS	NS	NS	NS	NS	NS	NS
1.28	A/B	NS	NS	NS	NS	NS	NS	NS	NS	NS	NS	NS	NS	NS	NS	NS	NS

**Table 2:** Cutting parameters.

Samples	Cuttingspeed (r/min)	Feed(mm)	Cuttingdepth (mm)	Machinetype	Sanding	Roughness(μm)
Smooth	500	0.21	0.2	Grinding	None	0.64
Rough	900	0.225	0.05	Turning	None	1.73
Rough + smooth	900	0.225	0.05	Turning	PEPA	1.42

**Table 3:** Feature parameters of a single cotton fiber.

Cotton	Micronaire	Moisture(%)	MaturityCoefficient	Upper Half MeanLength (mm)	Strength(g/tex)	Temperature(°C)
Combed	4.0	10	0.84	27.39	27.4	26.4
